# PXR Suppresses PPARα-Dependent *HMGCS2* Gene Transcription by Inhibiting the Interaction between PPARα and PGC1α

**DOI:** 10.3390/cells10123550

**Published:** 2021-12-16

**Authors:** Ryota Shizu, Kanako Ezaki, Takumi Sato, Ayaka Sugawara, Takuomi Hosaka, Takamitsu Sasaki, Kouichi Yoshinari

**Affiliations:** Laboratory of Molecular Toxicology, School of Pharmaceutical Sciences, University of Shizuoka, Shizuoka 422-8526, Japan; r_shizu@u-shizuoka-ken.ac.jp (R.S.); gp1537@u-shizuoka-ken.ac.jp (K.E.); s20803@u-shizuoka-ken.ac.jp (T.S.); m18143@u-shizuoka-ken.ac.jp (A.S.); hosaka@u-shizuoka-ken.ac.jp (T.H.); t-sasaki@u-shizuoka-ken.ac.jp (T.S.)

**Keywords:** PXR, PPARα, PGC1α, nuclear receptor, coactivator, gene transcription, drug–drug interaction, liver function

## Abstract

Background: PXR is a xenobiotic-responsive nuclear receptor that controls the expression of drug-metabolizing enzymes. Drug-induced activation of PXR sometimes causes drug–drug interactions due to the induced metabolism of co-administered drugs. Our group recently reported a possible drug–drug interaction mechanism via an interaction between the nuclear receptors CAR and PPARα. As CAR and PXR are structurally and functionally related receptors, we investigated possible crosstalk between PXR and PPARα. Methods: Human hepatocyte-like HepaRG cells were treated with various PXR ligands, and mRNA levels were determined by quantitative reverse transcription PCR. Reporter assays using the *HMGCS2* promoter containing a PPARα-binding motif and mammalian two-hybrid assays were performed in HepG2 or COS-1 cells. Results: Treatment with PXR activators reduced the mRNA levels of PPARα target genes in HepaRG cells. In reporter assays, PXR suppressed PPARα-dependent gene expression in HepG2 cells. In COS-1 cells, co-expression of PGC1α, a common coactivator of PPARα and PXR, enhanced PPARα-dependent gene transcription, which was clearly suppressed by PXR. Consistently, in mammalian two-hybrid assays, the interaction between PGC1α and PPARα was attenuated by ligand-activated PXR. Conclusion: The present results suggest that ligand-activated PXR suppresses PPARα-dependent gene expression by inhibiting PGC1α recruitment.

## 1. Introduction

Xenobiotic-sensing nuclear receptors, PXR and CAR, which are encoded by *NR1I2* and *NR1I3*, respectively, play crucial roles in the induction of drug-metabolizing enzymes and drug transporters in the liver [1,2]. These nuclear receptors share target genes and cooperate in the detoxification of harmful xenobiotics. The activation of these receptors by drugs or food ingredients results in the enhanced metabolism and excretion of co-administered drugs, so these receptors are mainly responsible for drug–drug interactions (DDIs) or drug-food interactions.

In addition to DDIs mediated by the induction of drug-metabolizing enzymes, our recent study revealed that CAR activation by antiepileptic drugs attenuated the fibrate-dependent expression of genes related to fatty acid oxidation and ketogenesis and decrease in blood triglyceride levels [3]. Mechanistic analyses demonstrated that CAR prevented fibrate-activated PPARα-mediated gene transcription by competing with the transcription coactivator PGC1α against PPARα [3].

The nuclear receptor PPARα regulates lipid metabolism in the liver [4]. In response to ligand binding, PPARα forms a heterodimer with RXRα and binds to the promoter sequences of its target genes related to lipid metabolism to induce their transcription. Fibrates are typical ligands of PPARα and induce the gene expression of PPARα target genes to stimulate lipid metabolism and lower blood triglyceride levels [5].

PXR also recruits PGC1α for gene transcription [6], so we hypothesized that PXR could functionally interact with PPARα through competition for PGC1α in modulating lipid metabolism. In fact, several reports suggest that PXR downregulates lipid metabolism in the liver and increases hepatic triglyceride levels. Treatment of mice with pregnenolone 16α-carbonitrile (PCN), a representative rodent PXR ligand, downregulated the hepatic mRNA levels of PPARα target genes and increased hepatic levels of triglycerides and cholesteryl esters [7]. PXR activation also downregulated the fasting-dependent expression of hepatic *Cpt1a* and *Hmgcs2*, which are PPARα target genes, and it increased hepatic triglyceride levels in mice [8]. In addition, our preliminary experiments suggested that PXR activation attenuates the interaction between PPARα and PGC1α [3]. Based on this background, we investigated the detailed molecular mechanism underlying the interaction between PXR and PPARα.

## 2. Materials and Methods

### 2.1. Materials

Bezafibrate, rifampicin, rifaximin, simvastatin, and SR12813 were purchased from Sigma-Aldrich (St. Louis, MO, USA). Oligonucleotides were commercially synthesized by Macrogen (Seoul, Korea). All other reagents were obtained from FUJIFILM Wako Pure Chemical (Osaka, Japan) or Sigma-Aldrich, unless otherwise indicated.

### 2.2. Plasmid Preparation

Human *HMGCS2* promoter-inserted pGL4.10 plasmids [3], human PPARα expression plasmid (hPPARα-pTargeT) [3], p3A4-pGL3 [6], and human PXR (hPXR) expression plasmid (hPXR-pTargeT) [6] were prepared previously. phRL-TK, phRL-CMV, phRL-SV40, pGL4.31, and PGC1α-expressing pFN21A plasmids were purchased from Promega (Madison, WI, USA). PGC1α-LXXLL-pFN11A—the pFN11A-based plasmid with the nuclear receptor-binding LXXLL motif of PGC1α (EAEEPSLLKKLLLAPANTQ)—and the pFN10A plasmid with hPPARα and hPXR cDNA were prepared as previously described (PPARα-pFN10A and PXR-pFN10A) [3,9]. hPXRΔAF2-pTargeT was produced using a KOD Plus mutagenesis kit (TOYOBO, Otsu, Japan) with specific primer sets for inserting a stop codon between Ala421 and Thr422.

### 2.3. Cell Culture

HepG2 and COS-1 cells (RIKEN BioResource Center, Tsukuba, Japan) were cultured in Dulbecco’s modified Eagle’s medium (DMEM) (FUJIFILM Wako Pure Chemical) supplemented with heat-inactivated 10% fetal bovine serum (GE Healthcare, Buckinghamshire, UK), nonessential amino acids (Thermo Fisher Scientific, Waltham, MA, USA), and antibiotic–antimycotic (Thermo Fisher Scientific). The cells were seeded in 96-well plates (BD Biosciences, Heidelberg, Germany) at 1 × 10^4^ cells/well. Twenty-four hours after seeding, plasmid transfection was performed.

HepaRG cells (Thermo Fisher Scientific) were cultured as described previously [3]. After a 72-h pre-culture, the cells were treated with drugs for 48 h and harvested for RNA extraction. Total RNA was subjected to quantitative reverse transcription PCR (qRT-PCR).

### 2.4. qRT-PCR

Total RNA was isolated using Sepasol RNA I (Nacalai Tesque, Kyoto, Japan). mRNA levels were measured as described previously [3].

### 2.5. Reporter Assays

Twenty-four hours after seeding, cells were co-transfected with reporter gene plasmid, expression plasmid, and *Renilla* luciferase-expressing plasmid using Lipofectamine 3000 (Invitrogen, Carlsbad, CA, USA) and treated with vehicle (0.1% or 0.2% dimethyl sulfoxide, DMSO) or drugs in serum-free DMEM for 24 h. Reporter activity was measured using the Dual-Luciferase Reporter Assay System (Promega) following the manufacturer’s instructions. Firefly luciferase luminescence was normalized to *Renilla* luciferase luminescence.

### 2.6. Mammalian Two-Hybrid Assay

Twenty-four hours after seeding, HepG2 cells were co-transfected with pGL4.31, PGC1α-LXXLL-pFN11A, and PXR- or PPARα-pFN10A using Lipofectamine 3000 and treated with vehicle (0.1% or 0.2% DMSO) or drugs in serum-free DMEM for 24 h. Reporter activity was measured using the Dual-Luciferase Reporter Assay System. Firefly luciferase luminescence was normalized to *Renilla* luciferase luminescence.

### 2.7. Statistical Analysis

Statistical analyses were conducted using GraphPad Prism version 9.3.0 (GraphPad Software, San Diego, CA, USA). Significance differences were assessed by Student’s *t*-test for the comparison of data from two groups and one-way ANOVA followed by Dunnett’s post hoc test or Bonferroni’s correction for the comparison of multiple group data, based on the experimental design. Statistical significance was set at *p* < 0.05, and asterisks indicate statistical significance. The values were not used for testing experimental hypotheses but were indicated to understand the differences between the compared groups. All experiments were repeated at least twice to confirm reproducibility. Sample sizes were specified before conducting experiments, and the number of experiments to check the reproducibility was determined after the initial results were obtained.

## 3. Results

### 3.1. PXR Ligand Treatment Downregulates PPARα Target Gene Expression

**Figure 1:**
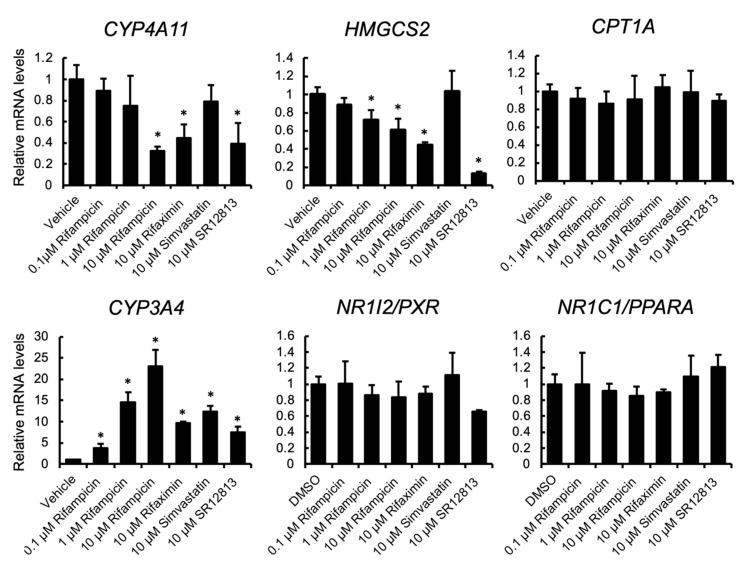
The influence of PXR ligands on gene expression in HepaRG cells. HepaRG cells were treated with rifampicin, rifaximin, simvastatin, SR12813, or vehicle (0.1% DMSO) at the indicated doses for 48 h. Total RNA was extracted and subjected to qRT-PCR. Data are shown as the mean ± S.D. (*n* = 4). Differences between vehicle-treated and drug-treated groups were assessed by Dunnett’s test (* *p* < 0.05).

To investigate the influence of treatment with PXR-activating drugs on PPARα target gene expression, human hepatocyte-like HepaRG cells were treated with rifampicin, rifaximin, simvastatin, or SR12813, and the mRNA levels were quantified (Figure 1). PXR activation was confirmed by the upregulation of the mRNA levels of *CYP3A4*, a representative PXR target gene. The mRNA levels of PPARα target genes, *HMGCS2* and *CYP4A11*, but not *CPT1A*, were downregulated by rifampicin treatment in a dose-dependent manner. The suppression was also observed with other PXR ligands, rifaximin and SR12813, but not simvastatin. *PXR* and *PPARA* mRNA levels were not affected by treatment.

**Figure 2:**
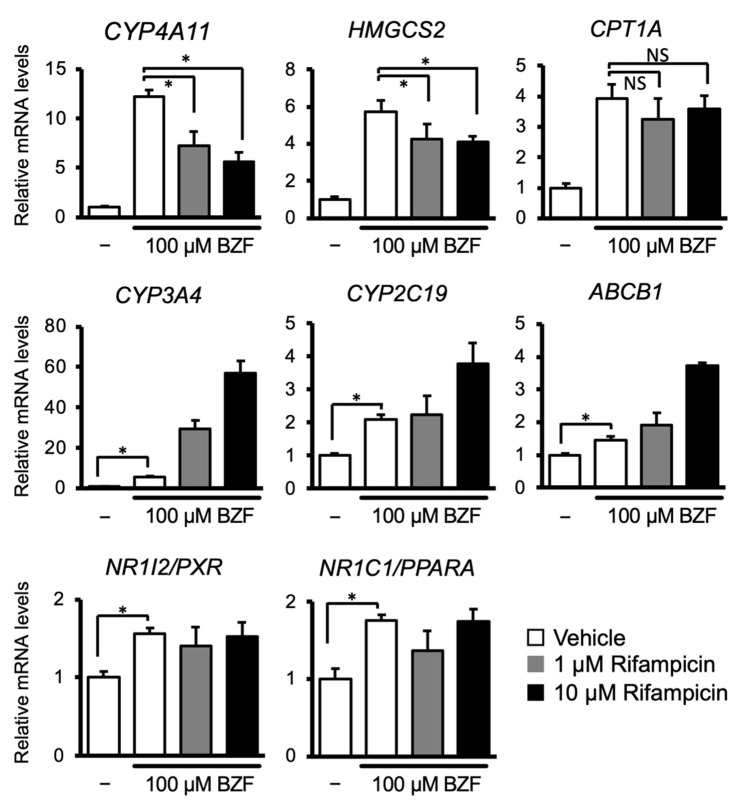
The influence of cotreatment with PPARα and PXR ligands on expression of their target gene. HepaRG cells were treated with 100 µM bezafibrate in combination with or without rifampicin (1 or 10 µM) for 48 h. Total RNA was extracted and subjected to qRT-PCR. Data are shown as the mean ± S.D. (*n* = 4). Differences between the indicated combinations were assessed using multiple paired *t*-tests with Bonferroni correction (* *p* < 0.05; NS, not significant).

Next, to investigate the interactions between PXR and PPARα, HepaRG cells were treated with the PPARα ligand, bezafibrate in combination with or without rifampicin, and the mRNA levels of the target genes of these nuclear receptors were quantified (Figure 2). As expected, bezafibrate treatment strongly upregulated the expression of *CYP4A11*, *HMGCS2*, and *CPT1A*. Rifampicin co-treatment suppressed bezafibrate-dependent upregulation of *CYP4A11* and *HMGCS2*, but not *CPT1A*. In contrast, the mRNA levels of PXR target genes, namely, *CYP3A4*, *CYP2C19*, and *ABCB1*, were increased by bezafibrate treatment alone. Cotreatment with rifampicin increased these mRNA levels in a dose-dependent manner. *PXR* or *PPARA* mRNA levels were induced slightly by bezafibrate treatment, and rifampicin co-treatment did not affect them. These results suggest that there is crosstalk between PXR and PPARα in human hepatocytes, where PXR-activating drugs attenuate the fibrate-mediated activation of PPARα-dependent gene transcription.

### 3.2. PXR Suppresses the PPARα-Dependent Gene Transcription

**Figure 3:**
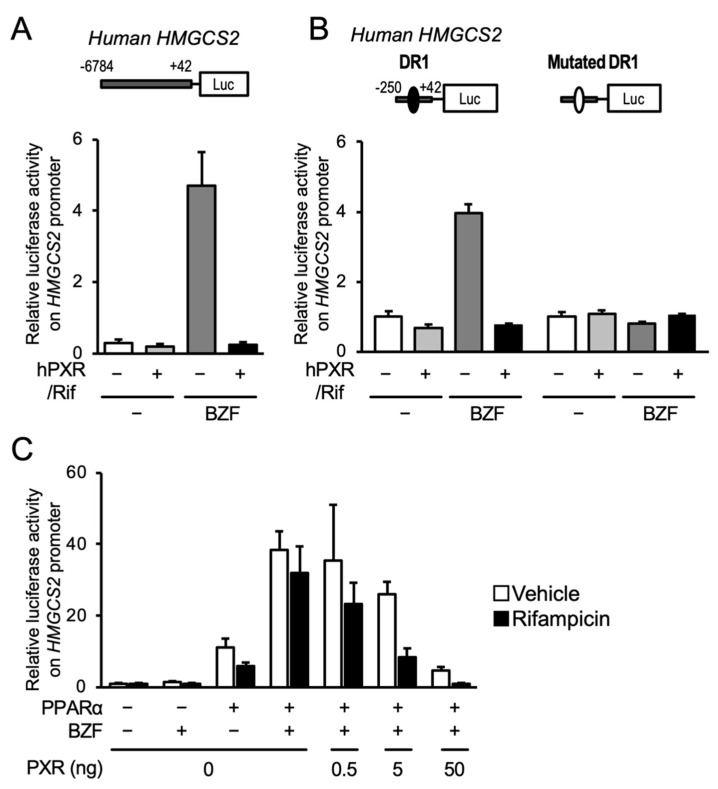
Influence of PXR activation on the PPARα-mediated reporter gene transcription under the control of the human *HMGCS2* promoter. (**A**) HepG2 cells were transfected with a reporter construct including −6784 to +42 of human *HMGCS2* in combination with or without a hPXR-expressing plasmid. The cells were treated with vehicle (0.2% DMSO), 100 µM bezafibrate (BZF), and/or 10 µM rifampicin (Rif) for 24 h, and reporter activity was determined. Data are shown as the mean ± S.D. (*n* = 4). (**B**) HepG2 cells were transfected with a hPXR expression plasmid and a reporter plasmid containing the −250 to +42 of human *HMGCS2*. The cells were then treated with vehicle (0.2% DMSO), 100 µM bezafibrate (BZF), and/or 10 µM rifampicin (Rif) for 24 h, and the reporter activity was determined. Closed and open circles in the plasmid diagrams represent the wild type and mutated DR1 motifs, respectively. Data are shown as the means ± S.D. (*n* = 4). (**C**) HepG2 cells were transfected with a hPPARα expression plasmid (10 ng) and/or hPXR expression plasmid (0.5, 5, or 50 ng) and a reporter plasmid containing the −250 to +42 of human *HMGCS2*. The cells were then treated with vehicle (0.2% DMSO), 100 µM bezafibrate (BZF), and/or 10 µM rifampicin for 24 h and the reporter activity was determined. Data are shown as the mean ± S.D. (*n* = 4).

To reveal the influence of PXR on PPARα-dependent gene transcription, we performed reporter gene assays in HepG2 cells, with *HMGCS2* as a model gene, using an expression plasmid for hPXR and a reporter plasmid containing −6784 to +42 from the transcription start site of human *HMGCS2* (Figure 3A). As expected, the *HMGCS2* promoter-driven luciferase reporter activity increased after bezafibrate treatment, and PXR expression and rifampicin treatment clearly prevented this effect. We previously demonstrated that PPARα controlled the transcription of *HMGCS2* via the DR1 motif in the proximal promoter [3]. Reporter assays were thus conducted with the proximal promoter sequence (−250 to +33) containing the DR1 motif, or the sequence with a mutation in the DR1 motif, which prevents PPARα from binding. Bezafibrate treatment increased reporter activity with the wild type construct, and PXR expression followed by rifampicin treatment completely prevented this increase (Figure 3B). Neither bezafibrate-dependent gene expression nor PXR-mediated repression was observed with the mutated DR1-containing plasmid. We further investigated the interaction using the PPARα expression plasmid and found that PPARα overexpression alone clearly increased reporter activity and that bezafibrate treatment further increased reporter activity (Figure 3C). Co-expression of PXR inhibited PPARα-mediated gene expression depending on the amount of PXR expressed. This PXR-mediated suppression was obvious in the presence of rifampicin. These results strongly suggest that ligand-activated PXR negatively regulates PPARα-dependent gene transcription.

### 3.3. PXR Competes with PPARα for PGC1α Binding

The coactivator PGC1α upregulated PPARα-dependent gene transcription and CAR attenuated PPARα-dependent gene transcription by competing with PGC1α [3], so we hypothesized that PGC1α competition is also involved in the PXR-dependent suppression of PPARα-dependent gene transcription.

**Figure 4:**
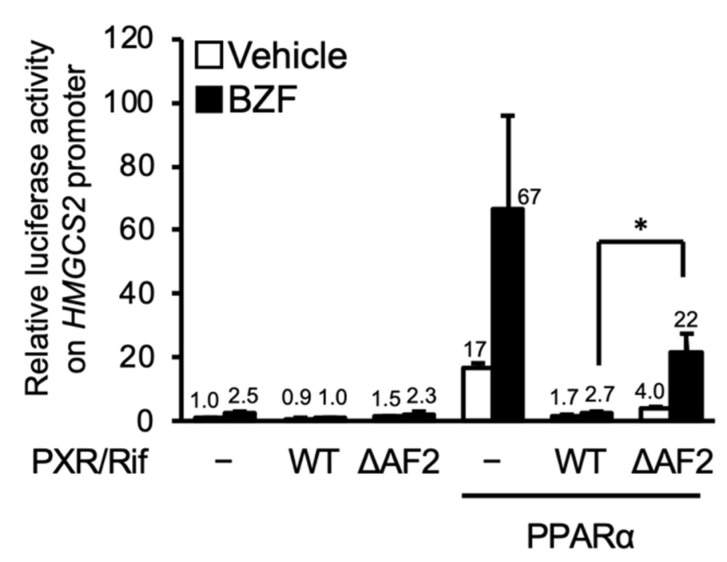
PXR-mediated suppression of PPARα-dependent reporter gene transcription. HepG2 cells were transfected with a reporter plasmid containing the −250 to +42 of human *HMGCS2*, expression plasmids for hPPARα, hPXR, and/or hPXR-ΔAF2 as indicated, and control phRL-SV40, followed by treatment with vehicle (0.2% DMSO), 100 μM bezafibrate (BZF), and/or 10 μM rifampicin (Rif) for 24 h. Relative reporter activity was determined. Data are shown as the mean ± S.D. (*n* = 4). * *p* < 0.05, Student’s *t*-test). The numbers above the bars indicate relative luciferase activity.

First, we investigated the role of AF2, which is a coactivator binding domain of nuclear receptors, in PXR-dependent suppression using a hPXR mutant lacking the AF2 domain (PXR-ΔAF2). As shown in Figure 4, PXR-mediated suppression was attenuated by deletion of AF2. These results suggest that interaction with a coactivator via the AF2 domain is involved in PXR-dependent inhibition of PPARα function.

**Figure 5:**
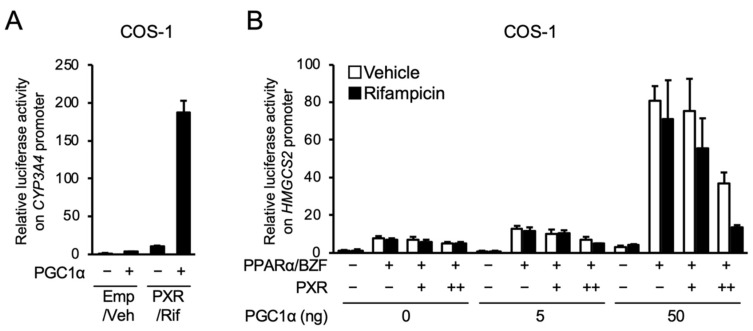
Influence of PGC1α overexpression on the PXR-dependent suppression of reporter gene transcription by PPARα in COS-1 cells. (**A**) COS-1 cells were transfected with p3A4-pGL3, expression plasmids for hPXR and/or PGC1α (50 ng) as indicated, and phRL-SV40, and treated with vehicle (Veh; 0.1% DMSO) or 10 μM rifampicin (Rif) for 24 h. (**B**) COS-1 cells were transfected with a reporter plasmid containing the −250 to +42 of human *HMGCS2*, expression plasmids for hPXR (1 or 10 ng), hPPARα (10 ng) and PGC1α as indicated, and phRL-SV40, and treated with vehicle (0.1% DMSO), 100 µM bezafibrate (BZF), and/or 10 µM rifampicin for 24 h. Relative reporter activity was determined. Data are shown as the mean ± S.D. (*n* = 4).

Next, we investigated the influence of PGC1α co-expression on PXR- and PPARα-dependent gene expression in COS-1 cells, in which PGC1α is expressed at lower levels than in HepG2 cells [3]. As shown in Figure 5A, co-expression of PGC1α potentiated the PXR-mediated expression of the reporter gene in the reporter assay with the promoter of *CYP3A4*, confirming the role of PGC1α as a coactivator in PXR-dependent gene transcription. Next, the influence of PGC1α expression on the PXR-PPARα interaction was investigated (Figure 5B). As expected, without PGC1α, PXR-mediated attenuation of PPARα-dependent gene transcription was not observed in COS-1 cells. In this cell line, PGC1α co-expression significantly enhanced the expression of the PPARα-dependent reporter gene, and PXR clearly inhibited the enhancing effects of PGC1α, which was more apparent in the presence of rifampicin. These results suggest that PGC1α is a key mediator of the PXR-PPARα interaction.

**Figure 6:**
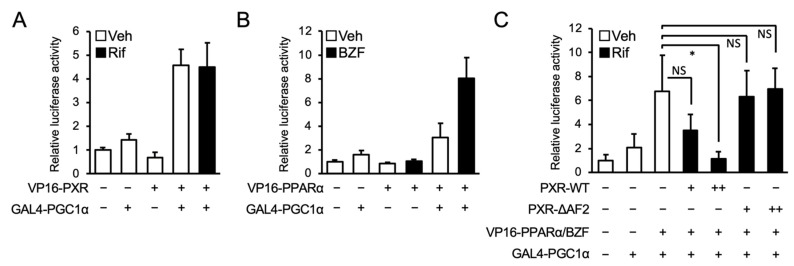
Influence of PXR on interaction between PPARα and PGC1α. (**A**,**B**) HepG2 cells were transfected with pGL4.31, PGC1α-LXXLL-pFN11A, an expression plasmid for hPXR (**A**) or hPPARα (**B**) fused to VP16 TAD. (**C**) An expression plasmid for hPXR or hPXR-ΔAF2 (1 or 10 ng) was co-expressed as in Figure 6b. Twenty-four hours after the transfection, the cells were treated with vehicle (Veh, 0.2% DMSO), 100 μM bezafibrate (BZF), and/or 10 μM rifampicin (Rif) for 24 h. Relative reporter activity was determined. Data are shown as the mean ± S.D. (*n* = 4). Differences between the indicated combinations were assessed by Dunnett’s test (* *p* < 0.05); NS, not significant.

Finally, a mammalian two-hybrid assay was conducted to investigate the interaction between PXR and PGC1α, using hPXR fused to the VP16 transactivation domain (TAD; VP16-PXR) and the nuclear receptor-interacting LXXLL motif of PGC1α fused to the GAL4 DNA-binding domain (DBD; GAL4-PGC1α). As indicated by an increase in luciferase activity, the interaction between PXR and PGC1α was confirmed (Figure 6A). In addition, we confirmed the strong interaction between PPARα and PGC1α in the presence of bezafibrate in assays with PPARα fused to the VP16 TAD (VP16-PPARα) and GAL4-PGC1α (Figure 6B). Next, to determine the influence of PXR expression on the interaction between PPARα and PGC1α, wild type PXR or the PXR mutant lacking AF2 (PXR-ΔAF2) was co-expressed (Figure 6C). As expected, the interaction was reduced by the expression of wild type PXR but not PXR-ΔAF2. These results suggest that PXR competes with PPARα for PGC1α binding.

## 4. Discussion

In this study, we tested the possibility of a functional interaction between PXR and PPARα in the liver, where both receptors are highly expressed. In human hepatocyte-like HepaRG cells, several PXR-activating drugs attenuated the expression of PPARα target genes. In a reporter assay using HepG2 cells and the promoter sequence of *HMGCS2* containing a PPARα binding motif, wild type PXR clearly prevented PPARα-dependent gene transcription, but no inhibition was observed with a PXR mutant lacking the coactivator-interacting AF2 domain, suggesting the involvement of coactivator interaction in the PXR-mediated suppression of transcription by PPARα. The results support involvement of coactivators by showing that the inhibition by PXR of PPARα-dependent gene transcription was not observed in COS-1 cells without PGC1α co-expression. Moreover, using a reporter assay with a PGC1α expression plasmid and a mammalian two-hybrid assay, we demonstrated that PPARα and PXR utilize PGC1α as a coactivator and compete for PGC1α binding. Taken together, these results imply that drug-induced activation of PXR may attenuate PPARα-dependent gene transcription by competing for PGC1α in human hepatocytes.

As PGC1α is a common coactivator not only for PXR and PPARα, but also for other nuclear receptors, competition for PGC1α binding might occur with other combinations of nuclear receptors. In fact, PXR is reported to compete for PGC1α binding to HNF4α and to downregulate HNF4α-mediated gene expression associated with hepatic cholesterol and glucose metabolism [10,11]. CAR has also been reported to compete for PGC1α binding with HNF4α [12]. In addition to PGC1α, various coactivators have been reported to contribute to transcription by nuclear receptors, including PXR and CAR [13]. Competition for nuclear receptor coactivator 1 (NCOA1, also known as SRC1) between PXR and HNF4α [14] and between CAR and PXR [15] has been reported. As nuclear receptors play pivotal roles in the regulation of various physiological functions and are pharmacological targets of many drugs, understanding the mutual regulation between nuclear receptors through coactivator competition may help to clarify the mechanism underlying the adverse effects of chemical compounds, such as pharmaceutical drugs.

In HepaRG cells, the mRNA levels of *CYP4A11* and *HMGCS2* were downregulated by PXR ligands, but *CPT1A* expression was not suppressed. The mechanism of these differences remains unclear at present, but this may be partly due to the differential contributions of multiple coactivators to the PPARα-dependent transcription of these genes. Although *CYP4A11*, *HMGCS2*, and *CPT1A* are well-known PPARα target genes [16], the extent of increase in their mRNA levels after bezafibrate treatment varied among the genes. These facts imply that a different factor(s) is involved in PPARα-induced transcription of these genes. One of the possible factors is that coactivators are recruited: PPARα bound to the *CYP4A11* or *HMGCS2* promoter and that bound to the *CPT1A* promoter may have different preferences for coactivators, and PPARα on the *CPT1A* promoter may recruit PGC1α to a lesser extent.

We previously demonstrated that CAR prevented the PPARα-dependent *HMGCS2* gene expression without binding to its promoter and affecting PPARα binding [3]. We thus expect that PXR may not bind to the *HMGCS2* promoter and downregulate its PPARα-mediated transcription without affecting the binding of PPARα to the promoter. It is possible that PXR also controls the PPARα-dependent gene transcription by preventing its binding to the PPARα-responsive element (PPRE) in the promoter of target genes. CAR is reported to prevent PPARα-dependent gene transcription not only by competing for PGC1α without affecting its DNA binding but also by directly binding to a PPRE in a target gene promoter [17]. Considering the similarity between CAR and PXR in their DNA binding domains with 66% amino acid identity [18], PXR may also occupy a PPRE to modulate PPARα-dependent gene expression. Further investigations are needed to elucidate the influence of PXR activation on PGC1α binding on DR1-bound PPARα in the *HMGCS2* promoter.

Although PXR activation attenuated the expression of PPARα target genes in HepaRG cells, PPARα activation induced the expression of PXR target genes. The results are unexpected considering that these receptors utilize and compete for common coactivators. It has been reported that the promoter of *PXR (NR1I2)* contains multiple binding sites for nuclear receptors, including PPARα, and PPARα significantly induced *PXR* transcription in a reporter assay with the *PXR* promoter [19]. In addition, treatment of rat primary hepatocytes with a PPARα ligand, such as WY-14643, or fasting, which activates PPARα, in mice was reported to increase *Pxr* mRNA levels [19,20]. Our results indicated that treatment with bezafibrate alone induced *PXR* mRNA levels as well as those of PXR target genes (Figure 2). In addition, PPARα has been reported to induce the transcription of PXR target genes *CYP3A4* and *CYP2C8* by directly binding to their promoters [21,22]. Given these findings, PPARα may directly upregulate the expression of PXR target genes or indirectly regulate it by increasing PXR expression independent of coactivator competition.

Several reports demonstrate the functional role of PXR in the regulation of hepatic lipid metabolism. For example, transgenic mice expressing hPXR in the liver showed hepatic triglyceride accumulation through decreased expression of β-oxidation-related genes and increased expression of the free fatty acid transporter CD36 [23]. On the other hand, PXR knockout mice showed resistance to high-fat diet intake-dependent upregulation of both hepatic and serum triglyceride levels [24]. PXR-dependent lipid accumulation was also observed in vitro, for example, in the liver cancer cell line HepG2 cells treated with rifampicin [25]. In a clinical study, treatment with rifampicin (600 mg daily for 1 week) was reported to elevate serum levels of triglycerides and cholesterols in patients [26] while rifampicin treatment (600 or 1200 mg daily for 14 days) had no effect on the serum triglycerides and cholesterols levels in another report [27]. Although rifampicin is not a rat PXR ligand its treatment also increased the plasma triglyceride levels in rats [28]. The role of PXR in the regulation of lipid metabolism remains controversial. In the present study, we have found the interaction between PXR and PPARα. As we focused on the influence of PXR activation the regulation of PPARα target genes in this study, we have not investigated its effects on lipid-related phenotypes and indicators. Further studies are thus needed to investigate the role of PXR in the regulation of hepatic lipid metabolism and to evaluate whether PXR inhibits the lipid-related functions of PPARα in human livers.

In conclusion, we have revealed a molecular mechanism for PXR-mediated suppression of PPARα-dependent gene transcription. This may be a novel mechanism for DDIs and/or drug–food interactions, because both receptors are activated by a number of pharmaceutical drugs, herbs, and food components. Investigation of such interactions in clinical situations in a future study will be of great interest for safe and efficient pharmacotherapy.

## Data Availability

The data presented in this study are available on request from the corresponding author.
